# The Patterns of Acquired Upper and Lower Extremity Amputation at a Tertiary Centre in Saudi Arabia

**DOI:** 10.7759/cureus.24026

**Published:** 2022-04-11

**Authors:** Fayez M Alshehri, Salwa A Ahmed, Sami Ullah, Haitham Ghazal, Shah Nawaz, Ahmed S Alzahrani

**Affiliations:** 1 Department of Physical Medicine and Rehabilitation, Rehabilitation Hospital, King Fahad Medical City, Riyadh, SAU; 2 Department of Physical Therapy, Rehabilitation Hospital, King Fahad Medical City, Riyadh, SAU; 3 Department of Rehabilitation Technology, Rehabilitation Hospital, King Fahad Medical City, Riyadh, SAU

**Keywords:** amputation program, prosthetics, infection, cancer, vascular disease, diabetes, trauma, rehabilitation, risk factors, amputations

## Abstract

Introduction: Upper and lower extremity amputations are associated with variable degrees of physical disability. In Saudi Arabia, disability still represents a major challenge for healthcare systems. There are insufficient data to describe the incidence and prevalence of impairment and disability. This study attempts to identify the patterns of limb amputations at a tertiary centre.

Methods: A retrospective chart review of the data of patients who received integrated tertiary healthcare in an amputation rehabilitation program (ARP) from 2013 to 2018 at King Fahad Medical City, Riyadh, Saudi Arabia was conducted. Data were collected using the demographic data and clinical history of amputees.

Results: A total of 412 patients were included in the study. Transtibial amputation (70%) and partial hand amputation (48%) were the most common levels for lower and upper limb amputations, respectively. There was a significantly higher rate of lower limb amputations secondary to vascular causes than that of upper limb amputations, which were more related to traumatic causes. Most patients, 213 (52%), were enrolled in an amputation rehabilitation program over a year after their amputation.

Conclusion: Vascular amputation is the most common cause of amputation. Most patients entered the rehabilitation program over a year after amputation. National guidelines for the prevention and management of the risk factors for vascular amputations should be developed.

## Introduction

Upper and lower extremity amputations are associated with psychological consequences and varying degrees of physical disability [1]. Amputation can be secondary to vascular compromise, trauma, cancer, infection, or congenital deformities. A 2015 study in the United States of America (USA) showed that around 1.7 million people had experienced the loss of a limb; this number is expected to double by 2050. [2]. Furthermore, around 185,000 new amputations occur annually in the USA. A study showed that the most common causes of limb loss were diabetes and peripheral vascular disease (PVD), followed by traumatic causes [3]. Another study from Australia described the age-standardized incidence of different levels of lower limb amputation in Australian hospitals between 2000 and 2010. During this period, the age-standardized incidence of lower limb amputation remained stable, with a significant gradual decline in transfemoral and transtibial amputations and increased partial foot amputation. This trend resulted from successful interventions for managing medical conditions leading to lower limb amputations [4]. In Europe, vascular amputation rates are relatively high compared with other causes of amputation [5]. However, a large study from South Korea between 1970 and June 1994, including 4258 amputees, revealed that the most common cause of amputation was trauma (66.7%), followed by PVD [6].

In Saudi Arabia, the lack of disability statistics still represents a significant challenge for healthcare systems. Furthermore, the lack of a National Amputation Registry to provide data describing the incidence and prevalence of limb amputation is another hindrance for healthcare policymakers. This has led to underestimating the actual burden of disability in the Kingdom of Saudi Arabia [7]. A study that extracted data from a 2016 Saudi national demographic survey showed that out of 20,064,970 citizens, 667,280 reported having disabilities [8]. Rehabilitation services for such people are provided by the ministry of health rehabilitation centers, other governmental sectors, and the private sector. The management of amputee patients requires a comprehensive, coordinated, interdisciplinary program throughout the continuum of care. The rehabilitation programs offer amputees full evaluation, management, and preventative measures for their medical problems. The latest practices in prosthetic and rehabilitation interventions allow them to achieve the highest level of function. A 14-year retrospective Saudi study published in the early 1990s analyzed all causes of amputation between 1977 and 1990 at the Riyadh Medical Rehabilitation Centre. The study results revealed that trauma was the leading cause of upper and lower limb amputations [9].

Recently, the incidence of limb amputations has declined in many countries. Moreover, the causes of amputation are mainly vascular. Limited data are available on the current common causes of amputation in the Kingdom of Saudi Arabia. This study attempts to identify the patterns and causes of upper and lower limb amputations.

## Materials and methods

This retrospective study investigated amputees receiving their prosthetic rehabilitation and follow-up in the amputation rehabilitation program (ARP) at the King Fahad Medical City (KFMC) between 2013 and 2018. An electronic chart review was conducted in the tertiary rehabilitation center in Riyadh, Saudi Arabia. The ARP is located at the rehabilitation hospital within KFMC. It was started in 2013 and is designed to help KFMC establish, maintain, and improve services for patients with upper and lower limb amputations. The program provides services for people who have suffered physical impairment due to amputations. The ARP accepts patients for both subacute inpatient and outpatient amputation rehabilitation services. It also receives referrals from all regions of the country, including internal transfers from the other hospitals within KFMC.

Data were collected using demographic characteristics and evaluation of amputation history, including age, region, date of amputation, amputation level, cause of amputation, date of enrollment in the rehabilitation program, and history of subsequent amputations. The inclusion criteria were adults and children of all ages who had undergone acquired upper or lower extremity amputations and were enrolled in the ARP. Non-Saudi patients and patients with incomplete data were excluded.

IRB approval was obtained from the KFMC institutional review board. The IRB approval number is 18-228. The analysis of the data was conducted using SPSS software (version 25, SPSS Inc, Chicago, Illinois, USA). Continuous variables were presented using the mean and standard deviation. A T-test was used to compare the means. The associations between categorical variables were explored using the chi-squared test. A P-value of less than 0.05 was considered significant.

## Results

**Figure 1:**
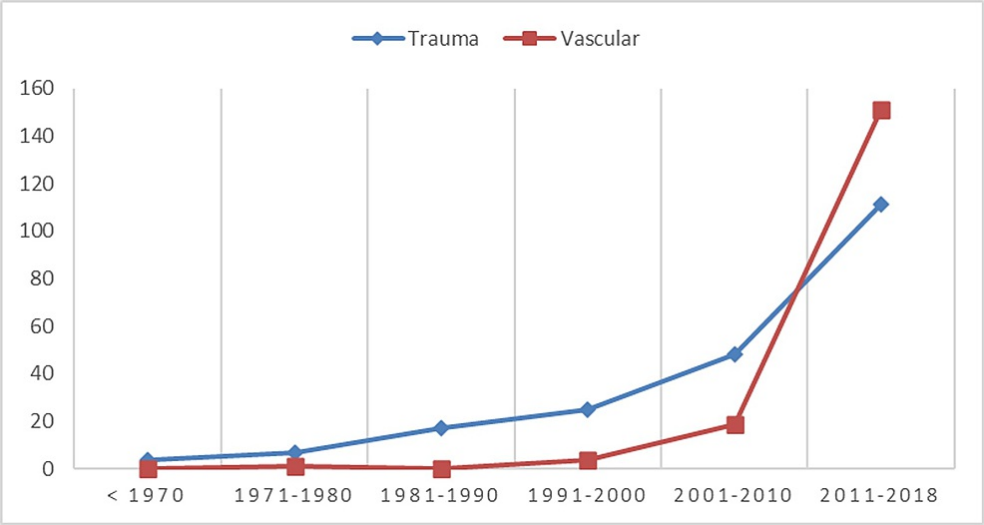
Trends in traumatic and vascular amputation cases

A total of 412 patients were included in the study. The mean age of the patients was 45.6 ± 19.9 years. There were 311 men and 101 women patients, a ratio of 3-1. Two hundred and eighty patients (68%) had their amputation between 2011 and 2018; 70 (17%), between 2001 and 2010; and the rest of the patients, 62 (15%), before 2001. In Figure 1, the trends of major limb amputation etiologies over the last five decades are shown.

**Figure 2:**
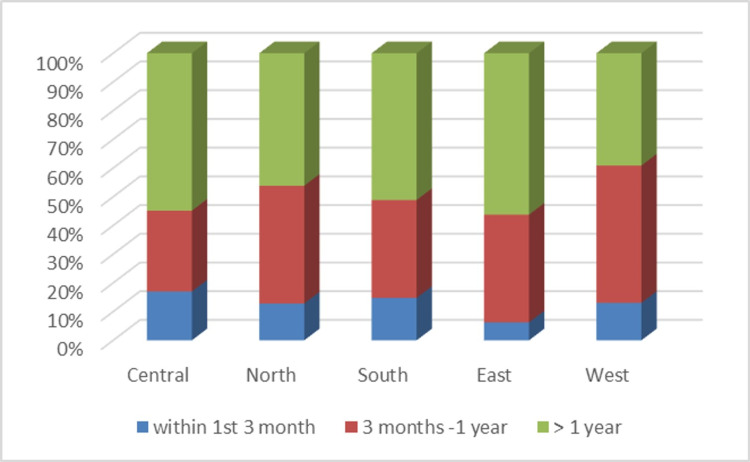
Interval between amputation and enrollment in rehabilitation by region

In total, 206 patients (50%) were from the central region, 78 (19%) from the northern region, 88 (21%) from the southern region, 16 (4%) from the eastern region, 23 (6%) from the western region, and one patient (0.2%) from outside Saudi Arabia. Most of the patients were from the central region, where the rehabilitation hospital is based. There were significantly more amputations in relation to regions (P = 0.005) and significantly more men involved than women (P = 0.04), including within each region (P = 0.03). Sixty-two patients (15%) were enrolled within three months of amputation, 137 (33%) were enrolled from three months to one year after amputation, and the majority, 213 (52%), were enrolled after one year. The variations in post-amputation rehabilitation between Saudi regions are shown in Figure 2.

There was a poor correlation between the region of residence and enrollment in rehabilitation (P = 0.548). There was also no statistically significant correlation between primary hospital region and enrollment in rehabilitation (P = 0.267).

Regarding limb involvement, 287 patients (70%) had unilateral lower limb amputation, 86 (21%) had unilateral upper limb amputation, 30 (7%) had bilateral lower limb amputation, one patient (0.2%) had bilateral upper limb amputation, and eight patients (2%) had amputations involving both upper and lower limbs. There was no statistically significant correlation between the level of amputation and region of residence (P = 0.401). The demographic characteristics of amputee patients are provided in Table 1.

**Table 1:** Demographic distribution of acquired upper and lower limb amputations (n = 412) ± SD: standard deviation, ARP: amputation rehabilitation program

Characteristics	Number mean ± SD	%
Gender		
Men	311	75.0
Women	101	25.0
Level of amputation		
Upper limb	86	20.9
Lower limb	287	69.7
Upper and lower limb	8	1.9
Bilateral upper	1	0.2
Bilateral lower	30	7.3
Right upper limb amputations		
Shoulder disarticulation	7	13.2
Trans-humeral	15	28.3
Elbow disarticulation	0	0
Trans-radial	8	15.1
Wrist disarticulation	1	1.9
Partial hand	22	41.5
Total	53	
Right lower limb amputations		
Hemipelvectomy	1	0.5
Hip disarticulation	3	1.6
Transfemoral	53	28.6
Knee disarticulation	5	2.7
Transtibial	91	49.19
Ankle disarticulation	1	0.5
Partial foot	31	16.8
Total	185	
Left upper limb amputations		
Shoulder disarticulation	6	12.2
Trans-humeral	13	26.5
Elbow disarticulation	1	2.0
Trans-radial	9	18.4
Wrist disarticulation	1	2.0
Partial hand	19	38.8
Total	49	
Left lower limb amputations		
Hemipelvectomy	1	0.6
Hip disarticulation	1	0.6
Transfemoral	43	24.3
Knee disarticulation	3	1.7
Transtibial	110	62.1
Ankle disarticulation	0	0
Partial foot	18	10.2
Total	177	
Cause of amputation		
Trauma	212	51.5
Tumor	20	4.9
Infection	5	1.2
Vascular	175	42.5
Subsequent amputation		
Nil	387	93.9
Within 5 years	23	5.6
>5 years	2	0.5
ARP enrolment		
Within 3 months	62	15.0
3 months to 1 year	137	33.3
>1 year	213	51.7

Partial hand amputation (40%) was the most common upper limb procedure, followed by amputation at the trans-humeral (27%) and trans-radial (17%) levels. Transtibial amputation (55%) was the most common level for lower limb amputation, followed by transfemoral (27%) and partial foot amputation (14%). There were significantly more lower limb amputations secondary to vascular causes and more upper limb amputations related to trauma (P < 0.001; Table 2).

**Table 2:** Patient characteristics and relation to causes of amputation (n = 412) The analysis of categorical variables related to amputee patients was done using Chi-square test. *For the age group at amputation in relation to cause of amputation (Chi-square value was 238.20 with degree of freedom of 12 and with P-value <0.001. **For the level of amputation in relation to cause of amputation (Chi-square value was 100.04 with degree of freedom of 12 and with P-value <0.001. ***For the subsequent amputation in relation to cause of amputation (Chi-square value was 31.23 with degree of freedom of 6 and with. P-value <0.001.

Variables	Causes of amputation, n (%)					P-value
	Total	Trauma	Tumor	Infection	Vascular	
Age at amputation						<0.001>*
1–14	76 (18.4)	65 (15.8)	2 (0.5)	3 (0.7)	6 (1.5)	
15–20	48 (11.7)	37 (9.0)	8 (1.9)	1 (0.2)	2 (0.5)	
21–40	105 (25.5)	83 (20.1)	4 (1.0)	1 (0.2)	17 (4.1)	
41–60	117 (28.4)	24 (5.8)	5 (1.2)	0	88 (21.4)	
>60	66 (16.0)	3 (0.7)	1 (0.2)	0	62 (15.0)	
Level of amputation						<0.001>**
Upper limb	86 (20.9)	80 (19.4)	2 (0.5)	1 (0.2)	3 (0.7)	
Lower limb	287 (70)	117 (28.4)	18 (4.4)	4 (1.0)	148 (35.9)	
Upper and lower limb	8 (1.9)	8 (1.9)	0	0	0	
Bilateral upper	1 (0.2)	1 (0.2)	0	0	0	
Bilateral lower	30 (7.3)	6 (1.5)	0	0	24 (5.8)	
Subsequent amputation						<0.001>***
Nil	387 (93.9)	211 (51.2)	20 (4.6)	5 (1.2)	151 (36.7)	
Within 5 years	23 (5.6)	1 (0.2)	0	0	22 (5.3)	
>5 years	2 (0.5)	0	0	0	2 (0.5)	

**Figure 3:**
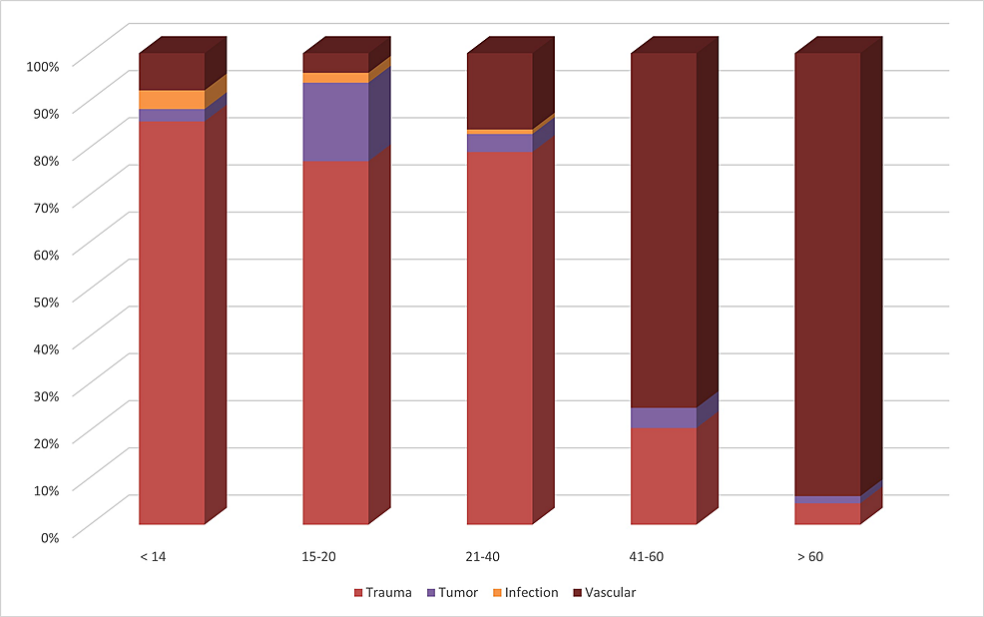
The relation between age group and cause of amputation

A total of 212 amputations of upper and lower limbs were secondary to trauma. There was a significantly higher rate of traumatic amputations for people under 40 years old and more vascular amputations for people over 40 years of age (P < 0.001) (Figure 3).

There was a significant, 12.57%, subsequent amputation within five years of the previous amputation, where it was secondary to PVD (P < 0.001; Table 2).

## Discussion

Over the last three decades, multiple studies investigating the etiologies of amputation have been published; however, only a few were from Saudi Arabia [9-16]. One study showed a higher rate of upper limb amputations than that of lower limb amputations for congenital limb deficiency [17]. The causes of acquired amputation were investigated by the first Saudi Arabia study published in the early 1990s. It was a 14-year retrospective analysis of all amputees between 1977 and 1990 at the Riyadh Rehabilitation Centre [9]. This study is the second to investigate the causes of amputation in a tertiary rehabilitation center since then. Between the 1970s and early 1990s, a Saudi study and a large study from South Korea described trauma as a major cause of upper and lower extremity amputations. Conversely, this study showed that the main causes of lower limb amputation were vascular, whereas for upper limb amputations, they were traumatic. Vascular causes were also more common above the age of 40, whereas trauma was more common under the age of 40. In a recent Saudi study at a major trauma center, 9.1% suffered amputation following motorcycle crashes, and lower limb amputation was more frequent than upper limb amputation [18]. In this study, amputations secondary to motorcycle injuries were only 1.7% of all causes of amputation, whereas 29.6% of amputations were secondary to motor vehicle accidents. The difference in motorcycle accident percentages between the major trauma center and our data is that the trauma center data were published in 2019 following the establishment of the trauma pathway.

Amputations secondary to cancerous tumors, however, represented only 4.9% of all amputations, whereas 43% resulted from vascular disease. A total of 172 patients (52.92%) had lower limb amputations for vascular causes. The most common medical conditions associated with lower-limb loss were diabetes and PVD, followed by trauma. One study identified hypertension, diabetes, obesity, and smoking as prevalent risk factors significantly associated with PVD, affecting mainly the lower limbs [19].

In this study, below-knee amputation showed a consistently high percentage with a high transtibial amputation level [20]. However, this study's decreased partial foot amputation percentage does not reflect its actual prevalence in other healthcare settings. Partial foot amputations are managed locally, or amputees do not seek prosthetic intervention [12]. A limitation of this study is that it included only the amputees who attended the ARP at the KFMC. Although, as a leading tertiary healthcare center, the ARP receives many referrals from throughout the Kingdom of Saudi Arabia; a significant proportion of amputees were not enrolled in this study.

In a large cohort study conducted in Saudi Arabia, it was noted that the country is facing a substantial rise in type II diabetes, with a prevalence among the top 10 countries worldwide. Moreover, the prevalence of diabetes-related morbidities is one of the highest worldwide, with 2.05% for foot ulcers and 1.06% for amputations. The overall survival rate has been reported as the worst among amputees compared with that of patients with diabetic foot ulcers [12,21]. In this study, the incidence of subsequent amputation due to PVD was 12.57% (P < 0.001) within five years from the initial or previous amputation. One study reported a 26% re-amputation rate secondary to vascular amputation in one year, with more than one-third of the patients dying within 12 months of their index amputation [22]. We were unable to get data on foot ulcers before the amputation as the patients were referred after the amputation. However, we recommend that our present data be upgraded to include diabetic foot ulcers with or without osteomyelitis, which will help identify regions that need to establish holistic diabetic care to prevent limb amputation.

The amputee population receives lifelong healthcare services based on their need to prevent complications, help them regain maximum independence levels, and achieve the highest quality of life. Amputations due to diabetic feet could be prevented through comprehensive approaches to diabetes control and the prevention of related complications by educating healthcare professionals and patients about foot care in diabetic patients [23]. In this study, out of the 175 vascular amputees involved in the ARP, 12.57% had a history of amputation within five years. It is difficult to draw conclusions about the effect of preventive measures before or after their enrolment because most of the patients were enrolled more than one year after their amputation and some were from various national regions. The ARP data revealed that only 15% of the 412 amputees were enrolled in the ARP within three months of amputation. The majority, 52%, were enrolled at least one year following their amputation. No correlation was found between the area of residence and the time of enrollment. Although the KFMC in Riyadh is part of the central region, only 16.9% of patients from this region enrolled within three months, and the majority, 54.9%, enrolled more than one-year post-amputation. This illustrates the lack of clear care pathways for amputees at the local and national levels.

Over the last four decades, huge changes have been observed in the Saudi lifestyle. Our study noted this concerning the recent epidemiological pattern of vascular and traumatic amputations. The rapid increase in socioeconomic development has been associated with improved living standards and technological advancements. Although life expectancy has increased from 72.5 to 75.0, from 1990 to 2010, for men and from 76.3 to 79.9 years for women, noncommunicable diseases and road traffic injuries are still the leading causes of death and disability locally [24]. Diabetes is also causing a financial burden for the Saudi healthcare system. The cost of healthcare services used due to diabetes and its related complications reportedly increased sevenfold between 2010 and 2020, according to a single conservative estimate [25]. That excludes the costly prosthetic component and rehabilitation provided by the government in Saudi Arabia.

In this review, there were few available epidemiological studies that investigated the extent of amputations in Saudi Arabia. Furthermore, the study data regarding amputation rehabilitation is not representative of the whole country. This study was conducted at a single tertiary healthcare center, which gives some indication of the causes of amputation; however, the results cannot be generalized because of possible regional factors. The lack of comprehensive rehabilitation services and the absence of a national amputation registry contribute to this study’s limitations. A national amputee registry, an amputation care pathway and the development of standards could improve the care provision on a larger scale.

## Conclusions

Vascular disease has become the most common cause of amputation in Saudi Arabia compared with three decades ago, mainly for patients over 40 years of age. However, trauma is still the main cause of amputation for people under 40 years of age. Transtibial is the most common level for lower limb amputation. The majority of the amputees enrolled in the prosthetic rehabilitation program were over a year post-amputation. Such delays can cause complications, and lead to increased costs and a negative impact on outcomes. A national registry, guidelines, and standards are required to enhance the prevention and management of complications and reduce the risk factors for vascular amputations. There should be more emphasis on legislation and public education to reduce trauma, which is the leading cause of amputation in younger age groups, mostly related to road traffic accidents.
